# Kanyawara Virus: A Novel Rhabdovirus Infecting Newly Discovered Nycteribiid Bat Flies Infesting Previously Unknown Pteropodid Bats in Uganda

**DOI:** 10.1038/s41598-017-05236-w

**Published:** 2017-07-13

**Authors:** Tony L. Goldberg, Andrew J. Bennett, Robert Kityo, Jens H. Kuhn, Colin A. Chapman

**Affiliations:** 1 Department of Pathobiological Sciences, University of Wisconsin-Madison, Madison, Wisconsin 53706 USA; 2 Global Health Institute, University of Wisconsin-Madison, Madison, Wisconsin 53706 USA; 3 Department of Zoology, Makerere University, Kampala, Uganda; 4 Integrated Research Facility at Fort Detrick, National Institute of Allergy and Infectious Diseases, National Institutes of Health, Frederick, Maryland 21702 USA; 5 Department of Anthropology and School of Environment, McGill University, Montreal, Quebec, H3A 2T7 Canada

## Abstract

Bats are natural reservoir hosts of highly virulent pathogens such as Marburg virus, Nipah virus, and SARS coronavirus. However, little is known about the role of bat ectoparasites in transmitting and maintaining such viruses. The intricate relationship between bats and their ectoparasites suggests that ectoparasites might serve as viral vectors, but evidence to date is scant. Bat flies, in particular, are highly specialized obligate hematophagous ectoparasites that incidentally bite humans. Using next-generation sequencing, we discovered a novel ledantevirus (mononegaviral family *Rhabdoviridae*, genus *Ledantevirus*) in nycteribiid bat flies infesting pteropodid bats in western Uganda. Mitochondrial DNA analyses revealed that both the bat flies and their bat hosts belong to putative new species. The coding-complete genome of the new virus, named Kanyawara virus (KYAV), is only distantly related to that of its closest known relative, Mount Elgon bat virus, and was found at high titers in bat flies but not in blood or on mucosal surfaces of host bats. Viral genome analysis indicates unusually low CpG dinucleotide depletion in KYAV compared to other ledanteviruses and rhabdovirus groups, with KYAV displaying values similar to rhabdoviruses of arthropods. Our findings highlight the possibility of a yet-to-be-discovered diversity of potentially pathogenic viruses in bat ectoparasites.

## Introduction

Bats (order Chiroptera) represent the second largest order of mammals after rodents (order Rodentia). Classically, bats are divided into two suborders: megabats (Megachiroptera) and microbats (Microchiroptera). Megabats, also referred to as fruit bats, are assigned to a single family, Pteropidae, whereas microbats are taxonomically more diverse^1^. Both megabats and microbats host numerous, taxonomically diverse viruses. Examples of megabat-borne viruses that are highly virulent for humans are Marburg virus and Nipah virus. Severe acute respiratory syndrome coronavirus is an infamous example of a microbat-transmitted human pathogen^1^. Consequently, characterization of bats, their viromes, and cross-species transmission of bat-borne viruses have become research priorities.

Much less effort has thus far been invested in understanding the role of bat ectoparasites in maintaining viruses in bat populations or potentially transmitting them to humans or mammals of other species. The high degree of specialization and diversity of certain bat ectoparasites suggests that they could, in fact, be reservoirs for certain viruses, maintaining them in their bat hosts. Alternatively, bats could be refractory to infection with ectoparasite viruses, but nevertheless these viruses could be infectious or even pathogenic for other mammals, including humans, and be transmitted through incidental bat ectoparasite bites.

Bat flies are eyeless, wingless, hematophagous dipteran insects (Brachycera: Muscomorpha: Hippoboscoidea) that are obligate bat ectoparasites with off-host breeding life stages. They are assigned to two families, the monophyletic Nycteribiidae and the probably paraphyletic Streblidae, and they infest bats throughout the Old and New Worlds^2, 3^. Bat flies of each family have evolved exquisite morphological and behavioral adaptations to life on bats, reflecting a long history of co-evolution^2^. Bat flies host a diverse community of bacteria, including bartonellae, some of which are zoonotic^4, 5^. Bat flies also vector hemosporidian parasites (*Plasmodiidae*: *Polychromophilus melaniferus*) that cause “bat malaria”^6^. On the other hand, evidence for a role of bat flies as reservoirs or vectors of viruses is scant. Only two viruses have been unambiguously identified in bat flies: the putative orthoreovirus Mahlapitsi virus and the putative orthobunyavirus Wolkberg virus, which were both found in the nycteribiid *Eucampsipoda africana* on pteropodid Egyptian rousettes (*Rousettus aegyptiacus*)^7, 8^. In addition, rhabdovirus RNA-like sequences were detected in nycteribiids and bats in Spain, but the sequences were too short (108 nt) to unambiguously substantiate virus infection^9^.

Here, we report the discovery and coding-complete genome sequence of a novel rhabdovirus, Kanyawara virus (KYAV), in a previously unknown nycteribiid bat fly collected from an unclassified megabat in western Uganda. Phylogenetic and genomic analyses of KYAV and its relatives offer new insights into the evolutionary and ecological associations of rhabdoviruses with both bats and arthropods.

## Results

**Figure 1:**
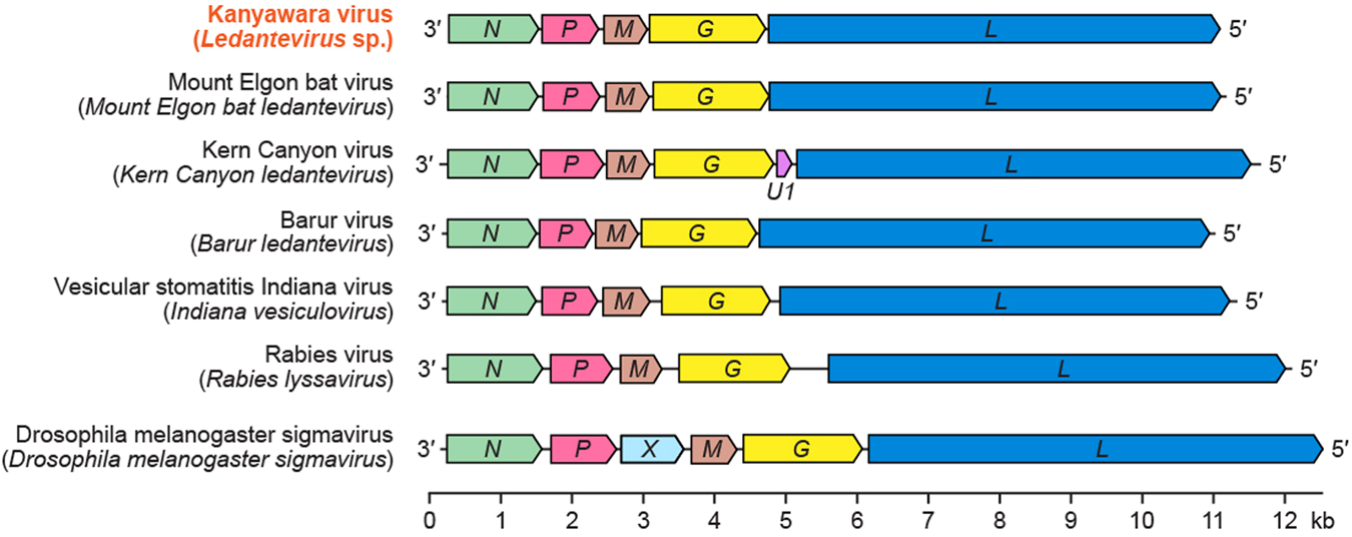
Comparison of the genome organization of Kanyawara virus (KYAV) to that of other members of the mononegaviral family *Rhabdoviridae*. Arrows signify open reading frames. Letters indicate the canonical rhabdovirus nucleoprotein (*N*), phosphoprotein (*P*), matrix (*M*), glycoprotein (*G*) and polymerase (*L*) genes and genus-specific open reading frames (*U1*, *X*).

Bat flies were found on six of nine pteropodid bats trapped at the edge of Kibale National Park, western Uganda, in 2010. Next-generation sequencing (NGS) of bat flies yielded 0.11 × 10^6^ to 1.59 × 10^6^ reads per sample. After quality trimming, rhabdovirus-like sequences were detected in five bat flies, each from a different bat. These sequences mapped with low similarity to conserved regions of rhabdovirus genomes (order *Mononegavirales*, family *Rhabdoviridae*). *De novo* assembly yielded a contiguous sequence of 10,843 nt in one bat fly sample (MPK004), with five open reading frames matching the canonical rhabdovirus genome organization (Fig. 1)^10^. Subsequent analysis of bat fly reads mapped 448 to 206,726 individual reads to this sequence, yielding coding-complete genomes in three other bat fly samples. Rhabdovirus coding genome sequences from bat flies of individual bats were 99.9% and 99.8% similar at the nucleotide and deduced amino acid levels, respectively. Viral read frequencies in the five positive bat fly samples ranged from 8,611 to 262,258 per million, with coverage ranging from 5-fold to 3,632-fold.

Sequencing of sera from the bats on which the bat flies were found yielded 1.17 × 10^6^ to 2.97 × 10^6^ reads per sample, but no reads mapped to the detected rhabdovirus genome. Application of this method at this sequencing depth is approximately as sensitive as real-time quantitative PCR^11^; therefore, bat sera could confidently be classified as negative for the virus. For further confirmation, however, we also tested all bat sera by PCR, and results were congruent with NGS results *(i*.*e*., all bat sera tested negative for the new rhabdovirus). Oral and urogenital swab samples from all bats also tested negative for the new rhabdovirus by PCR.

**Figure 2:**
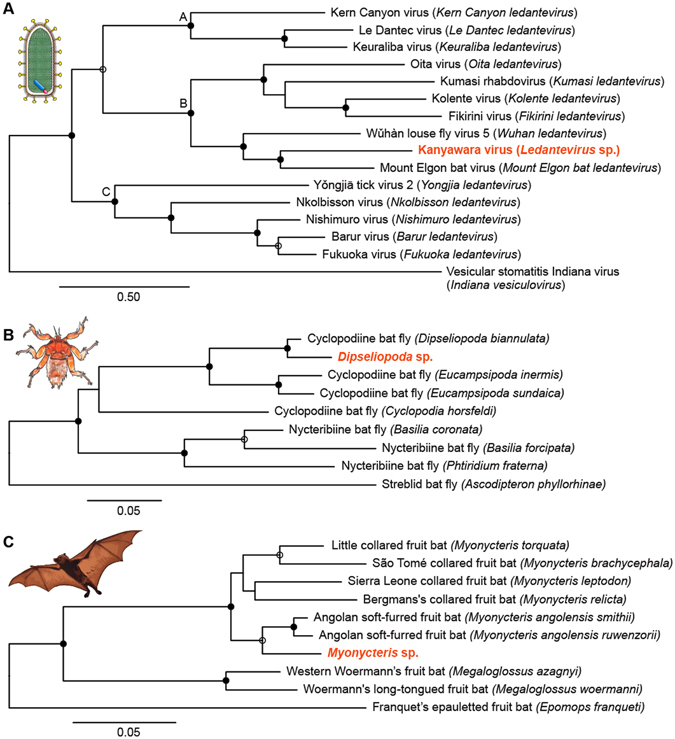
Maximum likelihood phylogenetic trees of rhabdoviruses (**A**), bat flies (**B**), and pteropodid bats (**C**). The rhabdovirus phylogeny is based on concatenated codon-based alignments (8,256 positions) of nucleotide sequences of the canonical rhabdovirus nucleoprotein (*N*), phosphoprotein (*P)*, matrix (*M*), glycoprotein (*G*), and RNA-dependent RNA polymerase (*L*) genes of 15 viruses of the subgroups A–C of the genus *Ledantevirus* with vesicular stomatitis Indiana virus (genus *Vesiculovirus*) as the outgroup. The bat fly phylogeny is based on concatenated codon-based alignments (876 positions) of mitochondrial cytochrome oxidase subunit II (COII) and cytochrome B (CYTB) nucleotide sequences of eight nycteribiids (subfamilies Cyclopodiinae and Nycteribiinae) with the streblid *Ascodipteron phyllorhinae* as the outgroup. The bat phylogeny is based on concatenated codon-based alignments (1,820 positions) of mitochondrial cytochrome C subunit I (COI) and CYTB nucleotide sequences of nine pteropodids of the subfamily Epomophorinae, with eight of them belonging to the tribe Myonycterini and Franquet’s epauletted fruit bats (tribe Epomophorini) as the outgroup. Circles on nodes indicate statistical confidence based on 1,000 bootstrap replicates of the data (closed circles = 100%; open circles ≥75%); scale bars indicate nucleotide substitutions per site.

Phylogenetic analysis (Fig. 2A; Supplementary Table S1) indicates the rhabdovirus to be a new member of the recently established genus *Ledantevirus* ^12, 13^. We named this virus Kanyawara virus (KYAV) after the village closest to the roost from which the bats were sampled. Sequence similarity between KYAV and other ledanteviruses based on concatenated, codon-based alignments of the canonical *N*, *P*, *G*, *M*, and *L* genes ranged from 62.4% (Mount Elgon bat virus) to 47.6% (Yngjiā tick virus 2) at the nucleotide level and from 59.3% (Mount Elgon bat virus) to 38.7% (Kern Canyon virus) at the deduced amino acid level, respectively. KYAV fulfills four of the five criteria of the International Committee on Taxonomy of Viruses (ICTV) *Rhabdoviridae* Study Group for classification in the genus *Ledantevirus*: A) the deduced amino acid sequence of the KYAV RNA-dependent RNA polymerase (L) diverges >7% from that of other ledanteviruses (KYAV:Mount Elgon bat virus = 35.2%); B) the deduced amino acid sequence of the KYAV glycoprotein (G) diverges 15% from that of other ledanteviruses (KYAV:Mount Elgon bat virus = 49.0%); C) KYAV has the same genome organization as other ledanteviruses (Fig. 1); and E) KYAV occupies a different ecological niche than other ledanteviruses. Criterium D (“can be distinguished in serological tests”) could not be evaluated due to the absence of a replicating KYAV isolate, but the high divergence of the sequence of KYAV *G*, the only ledantevirion surface protein, strongly suggests that KYAV is also serologically distinct^14^.

**Figure 3:**
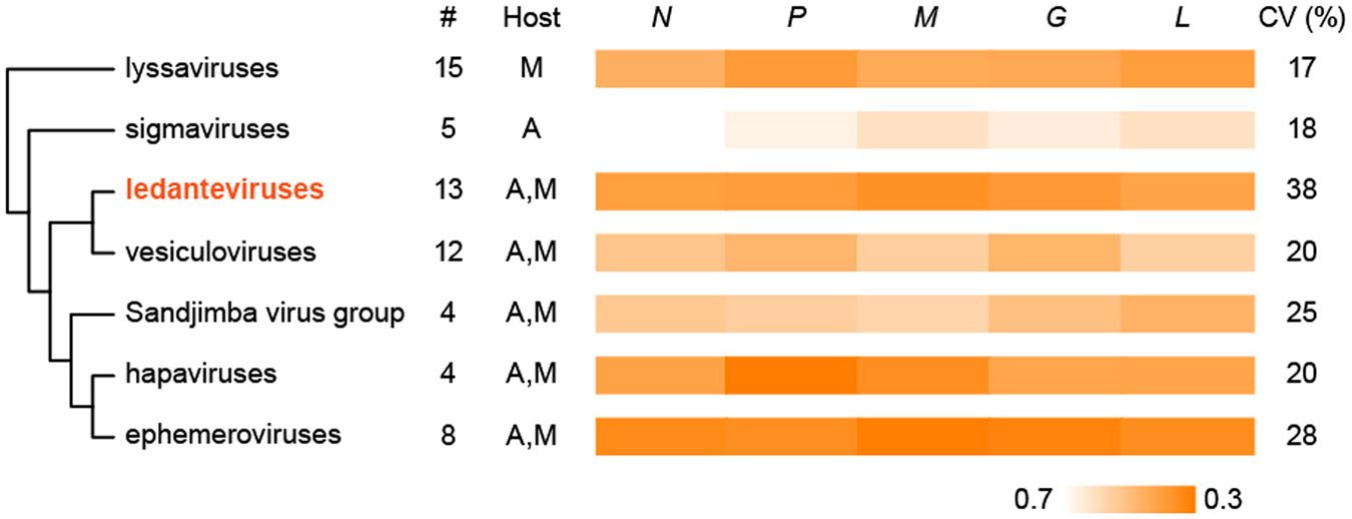
Relative dinucleotide frequency in seven rhabdovirus groups. Shading indicates relative CpG frequency of the canonical rhabdovirus nucleoprotein (*N*), phosphoprotein (*P*), matrix (*M*), glycoprotein (*G*) and polymerase (*L*) genes, averaged across viruses within each group. Scale (bottom) is inverted, so that darker colors indicate higher relative CpG depletion. Numbers indicate sample sizes of viruses per group, and letters indicate host associations of viruses within groups (A = arthropod; M = mammal). CV indicates the coefficient of variation (percent) of CpG across virus genomes within each group. The cladogram (left) shows non-metric evolutionary relationships among genera, based on^21^. Included in the analysis were all viruses with full *L*, *P*, *G*, *M* and *L* gene sequences available in GenBank and the Virus Pathogen Resource database^58^ as of December 12, 2016 (Supplementary Table S2).

An analysis of the CpG content of the KYAV genome and related rhabdoviruses revealed significant variation (analysis of variance [ANOVA] *F* = 11.443; 6 degrees of freedom; *P* <0.0001), with low relative CpG depletion in sigmaviruses, vesiculoviruses, and the Sandjimba virus group accounting for this trend (Holm *T*-statistic values ranging from 3.77 to 6.86; *P* values all <0.01; Supplementary Table S2). Figure 3 shows average CpG depletion by virus group and gene. CpG depletion was least pronounced for the insect-only sigmaviruses^15, 16^, but more pronounced in the mammal-specific lyssaviruses^17, 18^. These CpG variation patterns were generally consistent across the five canonical rhabdovirus genes *N*, *P*, *G*, *M*, and *L* within each virus group (Fig. 3). Within the genus *Ledantevirus*, KYAV and Oita virus have the lowest CpG depletion values (KYAV: 0.69; Oita virus: 0.72); these values were comparable to values for the insect-specific sigmaviruses (Supplementary Table S2). Variation in CpG frequency also differed significantly among rhabdovirus groups (Levine’s *W* statistic = 3.29; 6 degrees of freedom; *P* = 0.008). The coefficient of variation in CpG depletion was lowest for sigmaviruses and lyssaviruses and notably higher for the other virus groups (Fig. 3).

Phylogenetic analysis of mitochondrial DNA sequences from the collected bat flies revealed them to be members of the nycteribiid subfamily Cyclopodiinae, representing a putative new species of the genus *Dipseliopoda*. These sequences are approximately as divergent from bat flies of the most closely related cyclopodiine bat flies (*D*. *biannulatus*) as are the cyclopdiine bat flies of the species *Eucampsiopoda inermis* and *E*. *sundaica* (Fig. 2B).

Phylogenetic analyses of the sampled bats revealed them to be members of a putative new species, clustering as an outgroup to Angolan soft-furred bats (*Myonycteris angolensis*) and approximately as divergent from those bats as are bats of other species pairs within the genus *Myonycteris* (Fig. 2C).

## Discussion

Viruses of the family *Rhabdoviridae* infect vertebrates, invertebrates, and plants around the world^10, 19^. Their broad host range and wide geographic distribution reflect a deep evolutionary history of lineage-specific adaptation to particular host assemblages and ecologies of transmission^10, 19–21^. Bats are disproportionately represented among mammalian hosts of rhabdoviruses^18, 19^. For example, many viruses of the rhabdoviral genus *Lyssavirus*, including rabies virus, cause bat-borne zoonoses^18, 19^, and bats are the dominant vertebrate hosts for at least two of the three subgroups of the genus *Ledantevirus* ^10, 12^. The reasons for this association are not clear but may reflect the unique diversity, biology, or social systems of bats^1, 22, 23^.

Viruses of the family *Rhabdoviridae* also have deep evolutionary relationships with arthropods, as do numerous viruses of other families within the order *Mononegavirales* ^24, 25^. These relationships are evident today in the strong ecological associations that many rhabdoviruses maintain with arthropods. Viruses of the genus *Sigmavirus*, for example, are transmitted only vertically among insects^20, 26^, whereas viruses of the genera *Ephemerovirus*, *Tibrovirus*, and *Vesiculovirus* may infect mammals but typically are vectored by biting midges, mosquitoes, sandflies, or ticks^10, 19^. Similarly, plant rhabdoviruses (genera *Cytorhabdovirus* and *Nucleorhabdovirus*) are vectored by aphids, leafhoppers, or plant hoppers, and even fish rhabdoviruses transmitted directly through water may have associations with arthropods^10, 19, 27^. Rhabdovirus genome fragment integration into genomes of arthropods belonging to widely divergent lineages also supports a long history of rhabdovirus-arthropod coevolution^19, 24, 28^.

Despite this family-wide dual adaptation to arthropods and bats, vector-borne transmission of bat-associated rhabdoviruses has proven difficult to confirm. For example, Binger *et al*. searched for the vector of Kumasi rhabdovirus by trapping 1,240 female mosquitoes of six genera close to a large transient breeding colony of African straw-colored fruit bats (*Eidolon helvum*) in Ghana. No infected mosquitoes were identified^29^.

KYAV is a new putative member of the rhabdoviral genus *Ledantevirus*, sorting within subgroup B, which contains bat-associated viruses (Fig. 2A)^12^. The discovery of KYAV in nycteribiid bat flies suggests that KYAV could be a vector-borne virus, with bat flies as vectors. However, we did not find KYAV in the blood or on mucosal surfaces of the bats from which the bat flies were collected. This negative finding may indicate limited or transient viremia in bats, as is characteristic of, for instance, rabies virus^30–32^; however, other rhabdoviruses have been recovered from mucosal surfaces of bats^9, 33^. Alternatively, KYAV may be an insect-specific virus that does not infect bats. The detection of KYAV in 5 out of 6 (83%) bat flies sampled is consistent with this notion because infection rates of arthropod vectors with vector-borne viruses tend to be much lower than this rate, typically below 10%^34^.

The relative CpG dinucleotide frequency in viral genomes varies widely among taxa^35^ and within virus groups^36^. CpG depletion has been used as an index of viral host adaptation^16, 37, 38^, although a recent study by Di Giallonardo *et al*. found the measure to be useful only for comparisons of higher taxonomic ranks such as Arthropoda compared to Vertebrata^36^. CpG frequencies in KYAV and related rhabdovirus genomes (Fig. 3) therefore likely reflect a combination of virological factors and host adaptation, with emphasis on the former^36^. In this light, our analyses show that CpG depletion was lowest among the insect-specific sigmaviruses. Genomes of lyssaviruses (including rabies virus), which are transmitted directly between mammals in the absence of arthropod vectors, had higher levels of CpG depletion^17, 18^. Genomes of ephemeroviruses, hapaviruses, and vesiculoviruses, which infect vertebrates but are vectored by arthropods, had levels of CpG depletion comparable to those of the mammal-specific lyssaviruses, if not somewhat higher. KYAV and Oita virus genomes stand out among ledantevirus genomes by having very high CpG frequencies, similar to insect-specific sigmaviruses. We again caution that dinucleotide composition appears to be shaped more by virus taxon than by host species^36^; however, this metric remains useful for comparing similar viruses that infect very different hosts (e.g., mammals versus arthropods)^16, 39, 40^.

The nycteribiid bat flies in which we found KYAV are representatives of a putative new cyclopodiine species within the genus *Dipseliopoda* (Fig. 2B)^2^. This assessment is currently based solely on the phylogeny created here from mitochondrial cytochrome oxidase subunit II and cytochrome C DNA sequence data. Formal classification of these bat flies will have to await morphological characterization and additional genetic analyses.

Likewise, at the time of sampling, we thought based on morphologic characteristics that the collected bats were Angolan soft-furred bats (*Myonycteris angolensis*, also known as *Lissonycteris angolensis* ^41^). However, our analysis of mitochondrial DNA sequences placed these bats as outgroup to Angolan soft-furred bats. They appear to be novel members of the Epomophorinae in the genus *Myonycteris* divergent enough to merit consideration as members of a separate species. This assessment is preliminary as it presently relies only on a mitochondrial cytochrome oxidase subunit I and cytochrome C phylogeny (Fig. 2C). Morphological characterization and additional genetic analyses will be required to confirm this taxonomy. Nevertheless, the discovery of a putative new pteropodid bat is surprising given that Kibale National Park is one of the most extensively researched forested areas in Africa^42, 43^.

Overall, our data demonstrate that our understanding of the diversity of megabats, their ectoparasites, and their viruses is still fragmented. Viruses of bats are diverse in part because bats themselves are taxonomically diverse^23, 44^. Therefore, identifying unknown taxa of megabats would be important for understanding the true diversity of their ectoparasites and associated viruses. Unfortunately, limited biological material and a remote field location in the present case precluded other desirable analyses, such as serologic assessment of bats or other mammals. Future studies using enzyme-linked immunosorbent assay (ELISA) or western blots targeting the major antigenic proteins of KYAV (likely N and/or G) might help elucidate the ecology of this virus in bats and animals of other species. Such studies may also resolve whether the absence of circulating KYAV in the tested bat sera reflects transient viremia, as we speculate, or lack of infection.

Bat flies occasionally bite people^2, 45^. Therefore, enigmatic cases of human infection with bat-associated rhabdoviruses may have resulted from incidental bites by bat flies or other bat-associated arthropods. For example, in 1969, Le Dantec virus infected a British dockworker who was bitten by an unidentified insect while unloading peanuts from a ship that had come from Nigeria^29^. Novel, divergent rhabdoviruses have also been found in apparently healthy people in Africa, suggesting unknown pathways of zoonotic transmission^46^. Our identification of an unknown rhabdovirus on unknown bat flies of unknown bats suggests further research on the diversity of these insects and their role as disease vectors might prove fruitful.

## Methods

In February 2010, nine pteropodid bats were mist-netted from an established roost of approximately 12 bats in a peridomestic structure (a storeroom behind a kitchen) at the edge of Kibale National Park, western Uganda^43, 47^. Blood, oral swabs, urogenital swabs, and ectoparasites were obtained from bats, and bats were immediately released thereafter. All protocols for animal and sample handling were approved in advance by the Uganda Wildlife Authority, the Uganda National Council for Science and Technology, Makerere University, McGill University, and the University of Wisconsin-Madison, and were performed in accordance with all relevant guidelines and regulations.

Bat flies (the only ectoparasites found on sampled bats) were kept separate by bat and were stored whole at −20 °C in RNAlater solution (Thermo Fisher Scientific, Inc., Waltham, MA, USA). Swab samples were also stored in RNAlater solution at −20 °C. Blood was separated by centrifugation into components for long-term storage at −80 °C. Single bat flies and swab tips were homogenized by bead beating using a portable homogenizer (Terralyzer; Zymo Research Corporation, Irvine, CA, USA). RNA was extracted from all sample types and converted to cDNA in the field using lyophilized reagents (RNA to cDNA EcoDry Premix, TaKaRa Bio USA Inc., Mountain View, CA, USA) and then converted into double-stranded cDNA (NEBNext Second Strand Synthesis Module, New England Biolabs, Ipswich, MA, USA). DNA was stabilized for long-term storage and transport to the USA at ambient temperature (DNAstable, Biomatrica, San Diego, CA, USA).

DNA was reconstituted, and libraries were prepared for NGS as previously described^48, 49^; this method is approximately as sensitive as real-time quantitative PCR for detecting viruses^11^. Briefly, DNA was purified using Agencourt Ampure XP beads (Beckman Coulter, Brea, CA, USA). Approximately 1 ng of DNA was prepared for sequencing on a MiSeq instrument (Illumina, San Diego, CA, USA) using the Nextera XT DNA sample preparation kit (Illumina). Sequence data were analyzed using CLC Genomics Workbench version 8.5 (CLC bio, Aarhus, Denmark). Low-quality bases were trimmed (phred quality score < 30), short reads (<75 bp) were discarded, and the remaining reads were subjected to *de novo* assembly. Assembled contiguous sequences (contigs) were analyzed for nucleotide- (blastn) and protein-level (blastx) similarity to known viruses in GenBank. All sequences generated in this study were deposited into GenBank. All sequences used for analyses and their accession numbers are listed in Supplementary Table S1.

Genetic similarity between Kanyawara virus (KYAV) and its relatives was assessed using pairwise sequence comparisons in the computer program MEGA7^50^. Maximum likelihood phylogenetic analyses were conducted on codon-based alignments of concatenated virus genes, with poorly aligned regions removed. Alignments were created using the MAFFT algorithm^51^ implemented in Translator X^52^, with the Gblocks algorithm^53^ applied to remove poorly aligned regions. Trees were constructed using the maximum likelihood method implemented in PhyML^54^, with best-fit models of molecular evolution estimated from the data using jModeltest^55^. Trees were displayed using FigTree^56^. The same phylogenetic methods were applied to mitochondrial gene sequences of bats (COI and CYTB) and bat flies (COII and CYTB nucleotide sequences) extracted from deep sequence data to investigate the taxonomy of sampled bats and bat flies.

Based on coding-complete virus genome sequences detected in the course of bioinformatics analysis (see Results), PCR primers KYAV-10368 (5′-GCGAACCCGACGATCATAGT-3′) and KYAV-10695 (5′-GCTGTGCATTCCAGTCTCCT-3′) were designed to amplify a 327-bp region of the KYAV RNA-dependent RNA polymerase (*L*) gene. PCR conditions were optimized using bat fly cDNA samples known to be positive and negative for KYAV infection by NGS. The optimized PCR was used to test cDNA extracted from swab samples. PCRs were conducted using the HiFi kit (Kappa Biosystems, Wilmington, MA, USA), with 40 cycles at 94 °C for 15 sec, 56 °C for 15 sec, and 72 °C for 15 sec. Amplicons were visualized on 2% agarose electrophoretic gels stained with ethidium bromide.

The relative frequency of CpG dinucleotide pairs was calculated for each of the canonical rhabdovirus genes (*N*, *P*, *G*, *M*, and *L*) from KYAV and related rhabdoviruses using the R Biostrings package^57^. Rhabdovirus sequences included in this analysis were selected objectively, using all coding-complete genomes from major rhabdovirus clades (genera) with at least four members available in GenBank and the Virus Pathogen Resource database^58^ as of December 12, 2016. Differences in mean CpG depletion among groups were evaluated for statistical significance using ANOVA, with pairwise differences between groups examined using the Holm *post-hoc* method, which adjustments for multiple testing^59^. Variances in CpG depletion among groups were compared using Levine’s test^60^. Statistical analyses were performed in the computer package R^61^.

### Data Availability

All data generated during the current study are available in GenBank (accession numbers KY385385-385392) or are included in this published article and its Supplementary Information files.

## Electronic supplementary material

Supplementary Information
