# Supplementary material for: Kanyawara Virus: A Novel Rhabdovirus Infecting Newly Discovered Nycteribiid Bat Flies Infesting Previously Unknown Pteropodid Bats in Uganda
Source: Sci Rep. 2017 Jul 13;7:5287. doi: 10.1038/s41598-017-05236-w (PMC5509700; doi:10.1038/s41598-017-05236-w)
Supplement: Supplementary file 1 — Supplementary Information [file 41598_2017_5236_MOESM1_ESM.pdf]

Supplementary information

Title: ***Kanyawara Virus: A Novel Rhabdovirus Infecting Newly Discovered Nycteribiid Bat Flies Infesting Previously Unknown Pteropodid Bats in Uganda***

Tony L. Goldberg[a,b,c,*], Andrew J. Bennett[a], Robert Kityo[c], Jens H. Kuhn[d], and Colin A. Chapman[c,e]

a. Department of Pathobiological Sciences, University of Wisconsin-Madison, Madison, Wisconsin 53706, USA
b. Global Health Institute, University of Wisconsin-Madison, Madison, Wisconsin 53706, USA
c. Department of Zoology, Makerere University, Kampala, Uganda
d. Integrated Research Facility at Fort Detrick, National Institute of Allergy and Infectious Diseases, National Institutes of Health, Frederick, Maryland 21702, USA
e. Department of Anthropology and School of Environment, McGill University, Montreal, Quebec H3A 2T7, Canada

[*]**Corresponding author**: Tony L. Goldberg, email: tony.goldberg@wisc.edu.

Supplementary information includes:

1. Table S1: Sequences used for analyses
2. Table S2: Relative CpG dinucleotide frequencies of 62 rhabdoviruses (*Mononegavirales*: *Rhabdoviridae*)

Table S1: Sequences used for analyses

Bats (Chiroptera: Megachiroptera: Pteropodidae)

Taxon	*Location*	*Year*	*Host*	*Accession numbers (COI, CYTB)*
Angolan soft-furred fruit bat (*Myonycteris angolensis ruwenzor*	Democratic Republic of the Cong	2009	N/A	JX282941, JX283240
Angolan soft-furred fruit bat (*Myonycteris angolensis smithii*)	Cote d'Ivoire	2009	N/A	JX282939, JX283228
Bergmans's collared fruit bat (*Myonycteris relicta*)	Tanzania	2001	N/A	JX282952, JX283220
Franquet's epauletted fruit bat (*Epomops franqueti*)	Gabon	2005	N/A	JX282938, JX283288
Little collared fruit bat (*Myonycteris torquata*)	Cameroon	2007	N/A	JX283142, JX283138
**Newly discovered bat (*Myonycteris*sp.)**	**Uganda**	**2010**	**N/A**	**KY385387, KY385388**
São Tomé collared fruit bat (*Myonycteris brachycephala*)	São Tomé and Principe	1989	N/A	JX282951, JX283222
Sierra Leone collared fruit bat (*Myonycteris leptodon*)	Côte d'Ivoire	2009	N/A	JX282958, JX283218
Western Woermann's fruit bat (*Megaloglossus azagnyi*)	Côte d'Ivoire	2009	N/A	JX282948, JX283271
Woermann's long-tongued fruit bat (*Megaloglossus woermanni*)	Cameroon	2007	N/A	JX282944, JX283247

Bat flies (Diptera: Hippoboscoidea)

Taxon	*Location*	*Year*	*Host*	*Accession numbers (COII, CYTB)*
Streblid bat fly (*Ascodipteron phyllorhinae*)	Malaysia	2003	Bicolored leaf-nosed bat (*Hipposideros bicolor*)	DQ133113, DQ133149
Cyclopodiine bat fly (*Cyclopodia horsfeldi*)	Malaysia	2004	Variable flying fox (*Pteropus hypomelanus*)	KF273808, KF273720
Cyclopodiine bat fly (*Dipseliopoda biannulata*)	Africa	unknown	unknown	DQ133109, DQ133145
Cyclopodiine bat fly (*Eucampsipoda inermis*)	Malaysia	2003	Lesser dawn bat (*Eonycteris spelaea*)	DQ133112, DQ133148
Cyclopodiine bat fly (*Eucampsipoda sundaica*)	Malaysia	2004	Large flying fox (*Pteropus vampyrus*)	KF273833, KF273736
**Newly discovered bat fly (*Dipseliopoda* sp.)**	**Uganda**	2010	**Newly discovered bat (*Myonycteris* sp.)**	**KY385385, KY385386**
Nycteribiine bat fly (*Basilia coronata*)	Malaysia	2004	Peters's myotis (*Myotis ater*)	DQ133107, DQ133143
Nycteribiine bat fly (*Basilia forcipata*)	United States of America	2003	Long-legged myotis (*Myotis volans*)	DQ133100, DQ133136
Nycteribiine bat fly (*Phtiridium fraterna*)	Malaysia	2004	Bicolored leaf-nosed bat (*Hipposideros bicolor*)	DQ133094, DQ133130

Rhabdoviruses *(Mononegavirales: Rhabdoviridae)*

Taxon	*Locatiion*	*Year*	*Host*	*Accession number (full coding genome)*
Barur virus (*Barur ledantevirus*)	India	1962	Roof rat (*Rattus rattus wroughtoni*)	KM204983
Fikirini virus (*Fikirini ledantevirus*)	Kenya	2011	Striped leaf-nosed bat (*Hipposideros vittatus*)	KC676792
Fukuoka virus (*Fukuoka ledantevirus*)	Japan	1982	Biting midge (*Culicoides punctatus*)	KM205001
Kern Canyon virus (*Kern Canyon ledantevirus*)	United States of America	1956	Yuma myotis (*Myotis yumanensis*)	KM204992
Keuraliba virus (*Keuraliba ledantevirus*)	Senegal	1968	Northern savanna gerbil (*Gerbilliscus kempi*)	KM205021
Kolente virus (*Kolente ledantevirus*)	Guinea	1985	Jones's leaf-nosed bat (*Hipposideros jonesi*) and tropical bont ticks (*Amblyomma variegatum*	KC984953
Kumasi rhabdovirus (*Kumasi ledantevirus*)	Ghana	2011	African straw-colored fruit bat (*Eidolon helvum*)	KJ179955
Le Dantec virus (*Le Dantec ledantevirus*)	Senegal	1965	Human (*Homo sapiens*)	KM205006
Mount Elgon bat virus (*Mount Elgon bat ledantevirus*)	Kenya	1964	Hildebrandt's horseshoe bat (*Rhinolophus hildebrandtii*)	KM205026
**Newly discovered Kanyawara virus (*Ledantevirus* sp.)**	**Uganda**	**2010**	**Newly discovered bat fly (*Dipseliopoda* sp.) or bat (*Myonycteris* sp.)**	**KY385390**
Nishimuro virus (*Nishimuro ledantevirus*)	Japan	2009	Wild boar (*Sus scrofa*)	AB609604
Nkolbisson virus (*Nkolbisson ledantevirus*)	Cameroon	1965	Mosquito (*Eretmapodites leucopus*)	KM205017
Oita virus (*Oita ledantevirus*)	Japan	1972	Little Japanese horseshoe bat (*Rhinolophus cornutus*)	KM204998
Vesicular stomatitis Indiana virus (*Indiana vesiculovirus*)	United States of America	1998	Horse (*Equus caballus*)	AF473864
Wǔhàn louse fly virus 5 (*Wuhan ledantevirus*)	China	2011	Louse fly (unidentified hippoboscid)	KM817654
Yǒngjiā tick virus 2 (*Yongjia ledantevirus*)	China	2013	East Asian mountain haemaphysalid tick (*Haemaphysalis hystricis*)	KM817662

Table S2: Relative CpG dinucleotide frequencies of 62 rhabdoviruses *(Mononegavirales*: *Rhabdoviridae)*

Virus	Genus or group	GenBank/RefSeq accession number	Relative CpG dinucleotide frequency (by gene)				
			N	*P*	*M*	*G*	*L*
Adelaide River virus	*Ephemerovirus*	NC028246	0.439	0.596	0.530	0.392	0.427
Berrimah virus	*Ephemerovirus*	NC025358	0.331	0.413	0.276	0.415	0.364
Bovine ephemeral fever virus	*Ephemerovirus*	NC002526	0.293	0.271	0.100	0.400	0.381
Kimberley virus	*Ephemerovirus*	NC025396	0.444	0.360	0.279	0.170	0.315
Koolpinyah virus	*Ephemerovirus*	NC028239	0.232	0.269	0.267	0.301	0.232
Kotonkan virus	*Ephemerovirus*	NC017714	0.279	0.423	0.161	0.399	0.359
Malakal virus	*Ephemerovirus*	NC025400	0.330	0.253	0.235	0.295	0.309
Obodhiang virus	*Ephemerovirus*	NC017685	0.388	0.323	0.458	0.241	0.381
Yata virus	*Ephemerovirus*	NC028241	0.322	0.276	0.416	0.235	0.363
Flanders virus	*Hapavirus*	NC028235	0.520	0.329	0.297	0.414	0.395
Hart Park virus	*Hapavirus*	KM205011	0.365	0.358	0.447	0.504	0.444
Ngaingan virus	*Hapavirus*	NC013955	0.358	0.302	0.488	0.358	0.403
Wongabel virus	*Hapavirus*	NC011639	0.428	0.184	0.180	0.425	0.454
Barur virus	*Ledantevirus*	KM204983	0.237	0.242	0.283	0.287	0.301
Fikirini bat rhabdovirus	*Ledantevirus*	NC025341	0.608	0.627	0.493	0.528	0.494
Fukuoka virus	*Ledantevirus*	KM205001	0.078	0.168	0.162	0.182	0.234
**Kanyawara virus**	**Unclassified ledantevirus**	**KY385390**	**0.698**	**0.417**	**0.447**	**0.541**	**0.555**
Kern Canyon virus	*Ledantevirus*	KM204992	0.176	0.239	0.185	0.347	0.332
Keuraliba virus	*Ledantevirus*	KM205021	0.330	0.408	0.462	0.254	0.398
Kolente virus	*Ledantevirus*	NC025342	0.538	0.680	0.709	0.579	0.542
Kumasi rhabdbovirus	*Ledantevirus*	NC028236	0.569	0.354	0.280	0.471	0.496
Le Dantec virus	*Ledantevirus*	KM205006	0.362	0.321	0.404	0.324	0.362
Mount Elgon bat virus	*Ledantevirus*	KM205026	0.333	0.500	0.143	0.382	0.418
Nishimuro virus	*Ledantevirus*	AB609604	0.237	0.247	0.303	0.260	0.287
Nkolbisson virus	*Ledantevirus*	KM205017	0.397	0.491	0.477	0.360	0.474
Oita virus	*Ledantevirus*	KM204998	0.719	0.472	0.382	0.507	0.536
Aravan virus	*Lyssavirus*	NC020808	0.420	0.395	0.417	0.494	0.396
Australian bat lyssavirus	*Lyssavirus*	NC003243	0.619	0.504	0.611	0.525	0.432
Bokeloh bat lyssavirus	*Lyssavirus*	NC025251	0.501	0.325	0.490	0.473	0.379
Duvenhage virus	*Lyssavirus*	NC020810	0.523	0.451	0.387	0.416	0.394
European bat lyssavirus 1	*Lyssavirus*	NC009527	0.372	0.353	0.344	0.319	0.418
European bat lyssavirus 2	*Lyssavirus*	NC009528	0.499	0.482	0.389	0.525	0.471
Ikoma lyssavirus	*Lyssavirus*	NC018629	0.539	0.403	0.415	0.286	0.320
Irkut virus	*Lyssavirus*	NC020809	0.325	0.399	0.424	0.468	0.393
Khujand lyssavirus	*Lyssavirus*	NC025385	0.545	0.507	0.450	0.469	0.388
Lagos bat virus	*Lyssavirus*	NC020807	0.411	0.262	0.446	0.416	0.421
Lyssavirus Ozernoe	Unclassified lyssavirus	NC025408	0.375	0.268	0.483	0.495	0.425
Mokola virus	*Lyssavirus*	NC006429	0.523	0.397	0.442	0.464	0.384
Rabies virus	*Lyssavirus*	NC001542	0.577	0.453	0.568	0.515	0.441
Shimoni bat virus	*Lyssavirus*	NC025365	0.276	0.315	0.383	0.333	0.329
West Caucasian bat virus	*Lyssavirus*	NC025377	0.399	0.368	0.399	0.388	0.410
Drosophila melanogaster sigmavirus	*Sigmavirus*	NC013135	0.783	0.589	0.607	0.506	0.539
Drosophila obscura sigmavirus	*Sigmavirus*	NC022580	0.690	0.651	0.351	0.541	0.512
Shāyáng fly virus 2	*Sigmavirus*	NC031215	0.612	0.632	0.927	0.671	0.669
Wǔhàn fly virus 2	*Sigmavirus*	NC031278	0.768	0.730	0.558	0.743	0.629
Wǔhàn house fly virus 1	*Sigmavirus*	NC031282	0.706	0.745	0.641	0.807	0.735
Niakha virus	*Sripuvirus* (Sandjimba virus group)	NC025405	0.383	0.543	0.307	0.338	0.433
Oak Vale virus	Unclassified (Sandjimba virus group)	NC025399	0.602	0.476	0.539	0.440	0.451
Sunguru virus	Unclassified (Sandjimba virus group)	NC025401	0.573	0.464	0.621	0.602	0.443
Walkabout Creek virus	Unclassified (Sandjimba virus group)	NC028232	0.596	0.735	0.847	0.668	0.552
Chandipura virus	*Vesiculovirus*	NC020805	0.582	0.435	0.574	0.415	0.596
Cocal virus	*Vesiculovirus*	NC028255	0.606	0.436	0.516	0.518	0.566
Isfahan virus	*Vesiculovirus*	NC020806	0.466	0.572	0.289	0.462	0.518
Jurona virus	*Vesiculovirus*	NC025392	0.608	0.489	0.473	0.427	0.543
Malpais Spring virus	*Vesiculovirus*	NC025364	0.453	0.688	0.573	0.534	0.563
Maraba virus	*Vesiculovirus*	NC025255	0.416	0.487	0.623	0.492	0.569
Morreton virus	*Vesiculovirus*	KM205007	0.498	0.351	0.505	0.527	0.567
Perinet virus	*Vesiculovirus*	NC025394	0.302	0.591	0.500	0.666	0.610
Vesicular stomatitis Alagoas virus	*Vesiculovirus*	NC025353	0.542	0.351	0.778	0.435	0.600
Vesicular stomatitis Indiana virus	*Vesiculovirus*	AF473866	0.571	0.472	0.599	0.249	0.510
Vesicular stomatitis New Jersey virus	*Vesiculovirus*	NC024473	0.584	0.389	0.529	0.340	0.443
Yug Bogdanovac virus	*Vesiculovirus*	NC025378	0.676	0.505	0.750	0.674	0.664
